# Immune–vascular mural cell interactions: consequences for immune cell trafficking, cerebral blood flow, and the blood–brain barrier

**DOI:** 10.1117/1.NPh.9.3.031914

**Published:** 2022-05-14

**Authors:** Anna Barkaway, David Attwell, Nils Korte

**Affiliations:** University College London, Department of Neuroscience, Physiology and Pharmacology, London, United Kingdom

**Keywords:** immune–vascular interactions, pericytes, leukocytes, cerebral blood flow, blood–brain barrier, waste clearance

## Abstract

Brain barriers are crucial sites for cerebral energy supply, waste removal, immune cell migration, and solute exchange, all of which maintain an appropriate environment for neuronal activity. At the capillary level, where the largest area of brain–vascular interface occurs, pericytes adjust cerebral blood flow (CBF) by regulating capillary diameter and maintain the blood–brain barrier (BBB) by suppressing endothelial cell (EC) transcytosis and inducing tight junction expression between ECs. Pericytes also limit the infiltration of circulating leukocytes into the brain where resident microglia confine brain injury and provide the first line of defence against invading pathogens. Brain “waste” is cleared across the BBB into the blood, phagocytosed by microglia and astrocytes, or removed by the flow of cerebrospinal fluid (CSF) through perivascular routes—a process driven by respiratory motion and the pulsation of the heart, arteriolar smooth muscle, and possibly pericytes. “Dirty” CSF exits the brain and is probably drained around olfactory nerve rootlets and via the dural meningeal lymphatic vessels and possibly the skull bone marrow. The brain is widely regarded as an immune-privileged organ because it is accessible to few antigen-primed leukocytes. Leukocytes enter the brain via the meninges, the BBB, and the blood-CSF barrier. Advances in genetic and imaging tools have revealed that neurological diseases significantly alter immune–brain barrier interactions in at least three ways: (1) the brain’s immune-privileged status is compromised when pericytes are lost or lymphatic vessels are dysregulated; (2) immune cells release vasoactive molecules to regulate CBF, modulate arteriole stiffness, and can plug and eliminate capillaries which impairs CBF and possibly waste clearance; and (3) immune–vascular interactions can make the BBB leaky via multiple mechanisms, thus aggravating the influx of undesirable substances and cells. Here, we review developments in these three areas and briefly discuss potential therapeutic avenues for restoring brain barrier functions.

## 1 Introduction

Brain bordering tissues comprise the glia limitans, blood–brain and blood-cerebrospinal fluid (CSF) barriers, meninges, and skull bone marrow. They allow communication between the brain and peripheral immune system, facilitate cerebral supply of oxygen, glucose, ions, and various regulatory molecules while promoting the efflux of metabolic waste and limiting the influx of neurotoxic molecules, pathogens, and leukocytes. Over the past 15 years, the proposed existence of the glymphatic system, (re)discovery of the meningeal lymphatics, and advances in our understanding of cerebral immunosurveillance, the blood–brain barrier (BBB), and blood supply have given important new insight into how the brain functions. The brain exhibits an immune-specialized status to minimize damage to its largely non-proliferating (and hence long-lived) cells, and therefore, leukocyte–vascular interactions and trafficking are tightly controlled. Neuroinflammation and microvascular dysfunction are hallmarks of various neurological diseases including, but not limited to, Alzheimer’s disease (AD), stroke, brain tumors, epilepsy, and multiple sclerosis (MS). Capillaries are the site of the greatest vascular resistance within the brain^1^ and provide the largest surface area for solute exchange.^2^ Control of capillary function is therefore crucial for regulating energy supply, BBB maintenance, and immune trafficking.

Following an overview of the brain vascular network and CSF transport routes, we will review recent advances in immune cell trafficking, immune modulation of cerebral blood flow (CBF), and BBB properties in various neurological diseases.

## 2 Overview of the Brain Vasculature and CSF Transport Routes

**Fig. 1:**
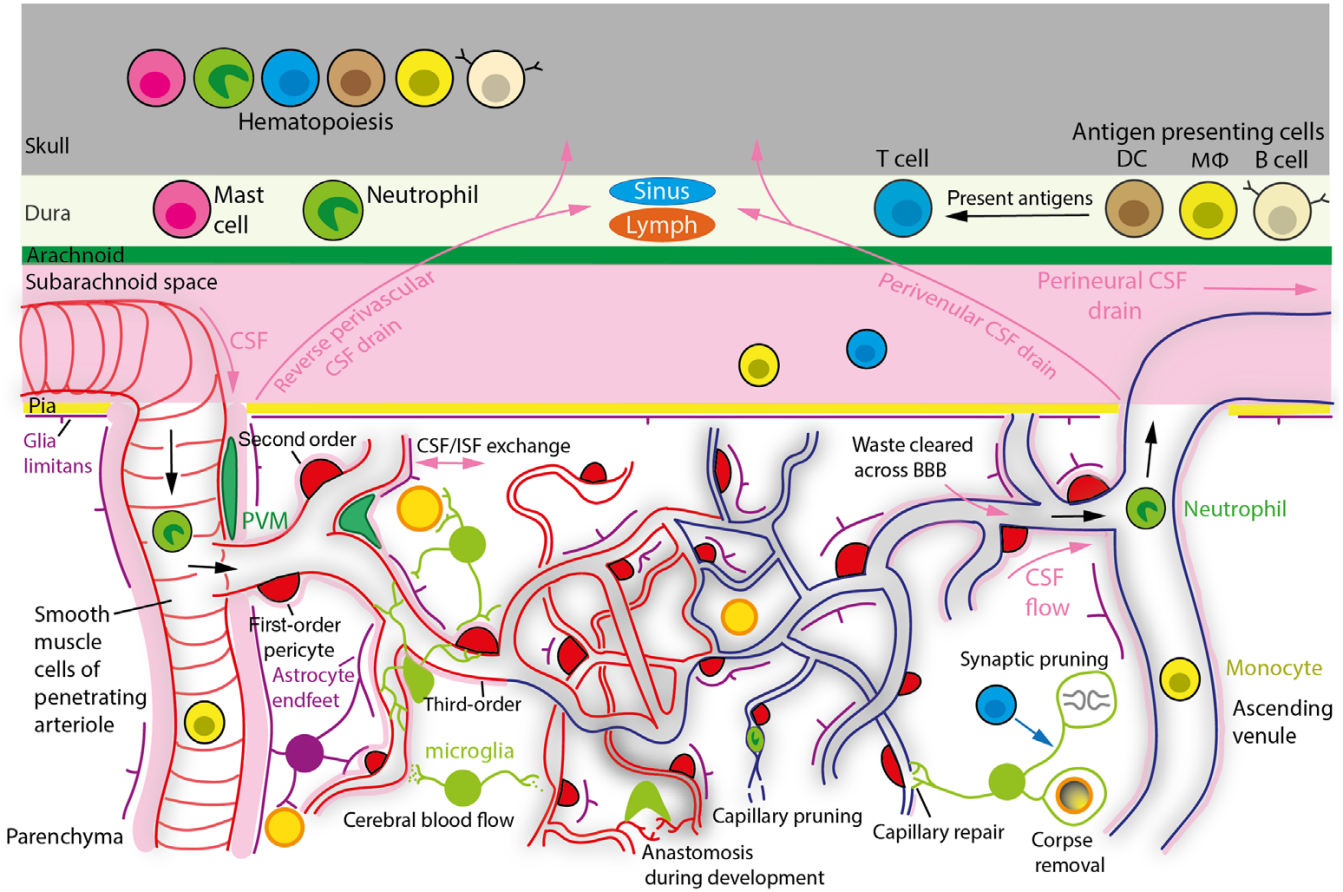
Organization of the brain vasculature, proposed waste clearance pathways and immune cells involved in brain surveillance. Blood in pial arteries enters the cerebral cortex parenchyma down PAs, goes through capillaries to supply oxygen and glucose to neurons, and exits the brain via AVs. Blood oxygenation decreases as it passes through the capillary bed, as indicated by the vessel color transitioning from red to blue. The PA is referred to as the zeroth-order vessel, the first branch off the arteriole as the first-order capillary, the branches of the first-order capillary as the second-order capillary and so on. Smooth muscle cells wrapping pial arteries and PAs, and pericytes with contractile processes around capillaries, control CBF by adjusting vessel diameter. CSF, which circulates in the subarachnoid space between the pia mater and arachnoid membrane, enters the perivascular space of PAs. Vessel pulsation driven by the heart, spontaneous vasomotion of SMCs and possibly first- to third-order capillary pericytes, creates a flow of CSF, which drains ISF waste either via reverse perivascular transport in small spaces of the PA wall, or through the brain via the glymphatic system (so called because AQP4 water channels in astroglial endfeet facilitate the exchange of CSF and ISF) possibly exiting via perivenular pathways to the subarachnoid space and dura. ISF might be drained (perineurally) along olfactory nerve rootlets into lymph vessels (not shown), via the dural meningeal lymphatics, across the BBB and/or venous sinuses into the blood and possibly through skull channels into the calvarial marrow. APCs (DCs, MΦ, and B cells) in the dura can capture CSF-derived brain antigens and present them to T cells, which modulate cognitive function by releasing neuromodulatory cytokines. The skull’s hematopoietic niche supplies leukocytes to the meninges. Cerebral waste molecules are also removed across ECs into the blood or phagocytosed by microglia and astrocytes. Microglia scan the brain for damage or infection related signals, prune synapses, at least in part by being programmed by T cells, regulate CBF, and NVC via purinergic signaling, repair damaged vessels by facilitating EC ligation and promote EC tip fusion during development, “anastomosis.” During aging, pericyte contraction and possibly neutrophil plugging (as occurs in AD) lead to a reduction of microvascular blood flow and may contribute to capillaries becoming pruned and phagocytosed by microglia.

Blood enters the cerebral cortex from its surface through penetrating arterioles (PAs), goes through a vast network of capillaries for brain–blood and blood–brain solute exchange, and exits via ascending venules (AVs) (Fig. 1).

CBF is controlled by smooth muscle cells (SMCs) around arteries and arterioles and by pericytes with contractile processes wrapping circumferentially around capillaries.^3^^–^^6^ Advances in tools to optogenetically depolarize and transgenically label pericytes, to image their vasomotor tone and intracellular ${\mathrm{Ca}}^{2+}$ (${\left[{\mathrm{Ca}}^{2+}\right]}_{i}$) activity *in vivo*, have revealed that pericytes throughout the capillary bed are contractile and that those of at least the first to third capillary branching order from PAs (where first-order refers to the first branch) contribute to neuronal activity evoked increases in CBF (Fig. 1).^7^^–^^13^ Pericytes are endowed with a wide range of ion channels allowing them to sense metabolic stimuli from the blood and brain to control CBF and solute transport appropriately (see Refs. 14–16 for transcriptome databases and Ref. 17 for a detailed review).

Respiratory motion, ciliary beating, vessel pulsation driven by the heart and spontaneous vasomotion of SMCs, and possibly pericytes of the first to third capillary branching order drive the flow of CSF, which facilitates brain buoyancy, waste clearance, and brain antigen presentation to systemic immune cells.^18^^–^^24^ CSF is generated by choroid plexus epithelial cells in the cerebral ventricles and circulates in the subarachnoid space from where it can enter the paravascular space of PAs (Fig. 1). Pulsations or spontaneous vasomotion move CSF either in a “reverse” manner back up the para-arterial spaces of PAs or through the brain via the postulated “glymphatic” system (involving water flow through the vascular endfeet of astrocytes), which drains cerebral interstitial fluid (ISF) possibly along AVs.^18^^,^^20^^,^^25^^–^^27^ This allows CSF to collect waste products and soluble antigens from the brain.

## 3 Immune Surveillance and Trafficking in the Brain: Pericytes as Immune Gatekeepers

### 3.1 Immune Surveillance

The brain is surveyed by microglia and astrocytes in the parenchyma, macrophages (MΦ) in the perivascular space, and by peripheral immune cells capturing brain antigens in the CSF and blood (Table 1).

**Table 1:** Types of immune cell discussed in this review.

Peripheral immune cells				
Cell type	Location	% of blood leukocytes	Numbers in blood ($10^{3}/\mu l$)	Main immunological role
Neutrophil	Peripheral blood	16* (mice)	1.4* (mice)	Recruited to sites of infection/injury to kill via phagocytosis, release of granular contents or via pathogen trapping in extracellular traps; usually the first peripheral cells recruited to inflamed tissues.
		60** (human)	3.4** (human)	
Monocyte	Peripheral blood	5* (mice)	0.4* (mice)	Patrolling phagocytic cells involved in clearance of debris, phagocytosis, capturing and killing microbes once recruited to tissues.
		5** (human)	0.26** (human)	
B lymphocyte	Peripheral blood	77* (mice)	Total lymphocyte count : 6.87* (mice)	Differentiate into plasma cells or memory B cells upon antigen recognition to produce antigen-specific antibodies for long-lasting immunity against secondary antigen challenges.
CD4+ T lymphocyte	Peripheral blood and CSF	27** (human)	1.52** (human)	Recognize major histocompatibility complex (MHC) class II on APCs and aid recruitment of other immune cell subsets.
CD8+ T lymphocyte	Peripheral blood and CSF			Recognize MHC class I and kill via secretion of cytokines, cytotoxic granules or by inducing apoptosis.
Major tissue immune cell types				
Cell type	Location	% of CNS leukocytes		Physiological role
PVM	Perivascular space, meninges, and choroid plexus	∼10 ***		Myeloid cells closely associated with the vasculature which scan the perivascular space, phagocytose debris and initiate recruitment of peripheral leukocytes.
Microglia	Brain parenchyma	∼80 ***		Resident immune cells of the brain that patrol the parenchyma, send out processes toward sites of injury to confine damage; scavenge foreign material, debris and synapses for removal; involved in synaptic pruning, neurogenesis and axonal growth.
DC	Meninges and choroid plexus	∼3 ***		Role in antigen capture, processing and presentation to T cells to propagate immune responses.
Mast cell	Meninges, choroid plexus, and parenchyma	∼0.5 ***		Derived from haematopoietic stem cells, long lived and resident cells, where they can interact with glia cells to orchestrate inflammation. Role not fully determined.

* 8–10 week old male mice.^28^

** Male adults.^29^

*** 2 month old C57BL6 mice as assessed by mass cytometry.^30^

**Fig. 2:**
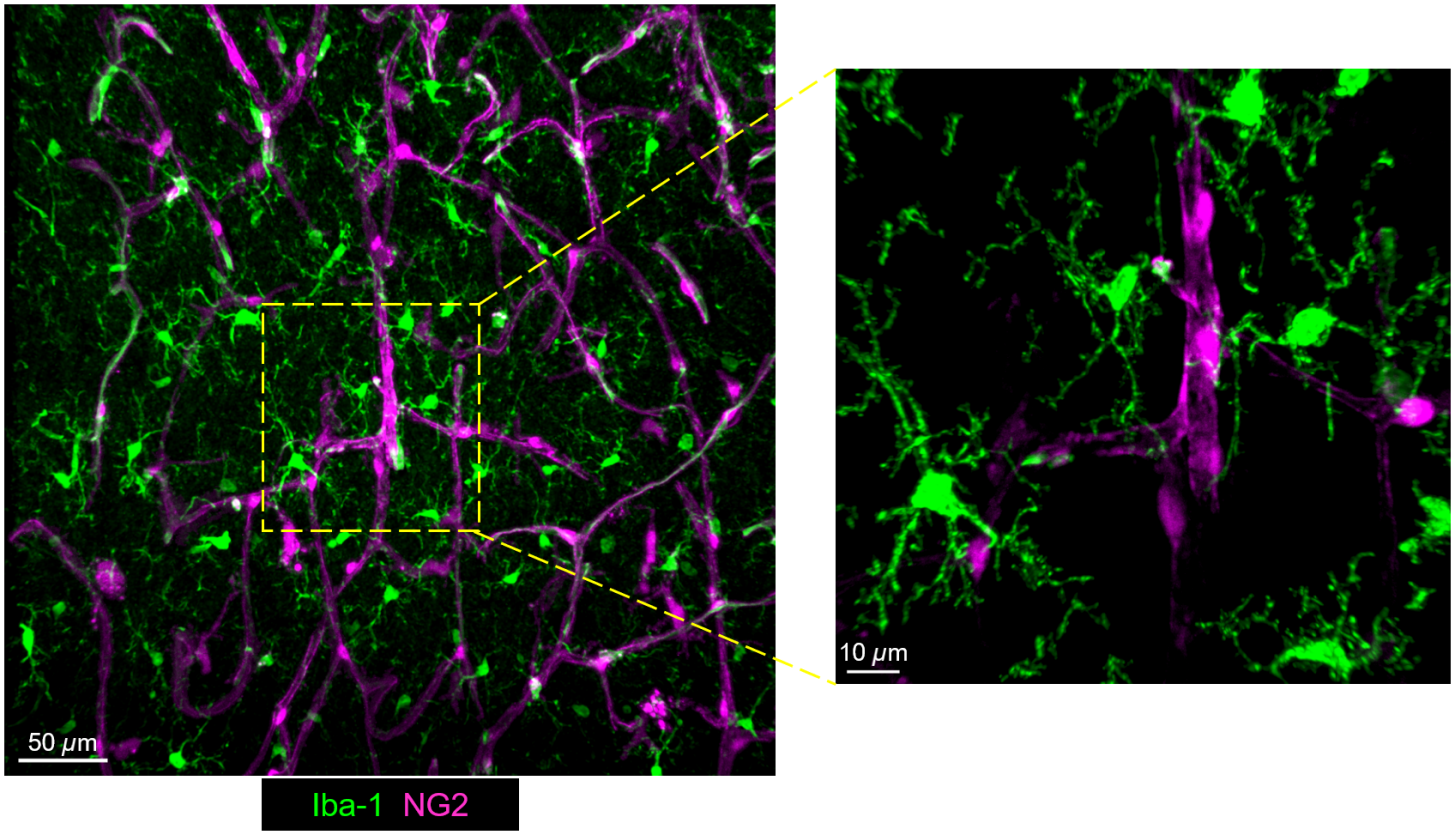
Microglia–vascular interactions in the murine cerebral cortex—confocal images from murine brain slices depicting the cortical parenchymal vascular bed. Microglia (green: labeled for Iba1) are often closely associated with the vasculature (magenta: pericytes visualized using NG2-DsRed transgenic mice) and have processes that can directly contact the abluminal vessel wall.

Microglia are highly ramified cells that constantly survey the brain parenchyma for infection or injury,^31^^–^^33^ phagocytose dead neurons,^34^ monitor and prune synapses,^35^^–^^37^ and regulate neuronal activity,^38^ in part by forming purinergic somatic junctions with neurons^39^ and also by releasing adenosine onto neuronal $A_{1}$ receptors.^40^ By extending and retracting their highly motile processes, microglia screen the entire brain parenchyma every few hours.^33^ A second mode of process motility called “chemotaxis” occurs when microglia send out processes toward sites of brain injury to confine brain damage, phagocytose cell debris, and limit secondary brain injury.^41^ Clearance of waste products is facilitated by tunnelling nanotubes connecting neighbouring microglia.^42^ Moreover, astrocytes, which are also capable of phagocytosis,^43^^–^^47^ occupy distinct phagocytic territories from those of microglia to aid in debris removal.^48^ Microglial immunophenotypic features can vary considerably across brain regions,^49^ highlighting their heterogenous nature. Microglia also closely associate with capillaries to modulate their functions (Fig. 2), as discussed in detail below.

**Fig. 3:**
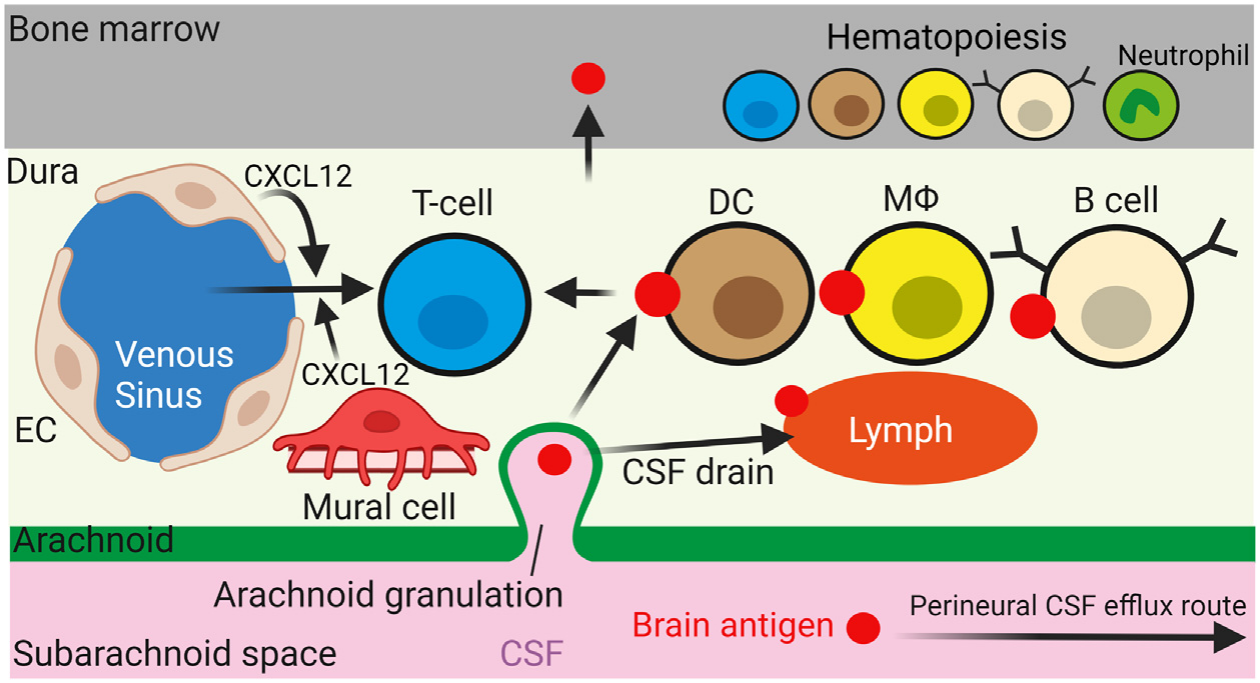
Dural immune cells survey brain antigens supplied by the draining CSF. CSF antigens may enter the dura through arachnoid granulations and become captured by APCs including DCs, MΦ, and possibly B cells. Endothelial (EC) or mural cell release of CXCL12 recruits T cells across the fenestrated endothelium of dural venous sinuses (on left), allowing T cells to survey antigens presented by APCs. CSF antigens are presented to immune cells in cervical lymph nodes (not shown) by being drained along perineural and dural lymphatic routes. Meningeal immune cells are repopulated by immune cells generated in the skull bone marrow. Figure created using BioRender.^54^

It is now evident that brain immune surveillance is not restricted to microglia, but also involves brain border-associated immune cells that have phenotypic properties distinct from microglia as revealed by recent high dimensional single-cell cytometry studies.^30^^,^^50^ Border-associated immune cells screen CSF-derived molecules in the perivascular spaces, meninges (dura, arachnoid, and pia), choroid plexus, deep cervical lymph nodes, and possibly the skull.^51^^,^^52^ CSF antigens drained from the brain can enter the dura, where antigen-presenting cells (APC) [including dendritic cells (DCs), MΦ, and B cells] capture brain antigens and present them to patrolling T cells^53^ (Fig. 3). In the dura, endothelial and mural cells (mainly pericytes and SMCs) recruit T cells by secreting the chemokine CXCL12, which binds to CXCR4 on T cells to promote their migration from the blood through the fenestrated endothelium of the dural venous sinuses.^55^^,^^56^ T cells modulate neuronal function probably by secreting cytokines from the meninges,^57^^,^^58^ or by migrating from the meninges into the brain, for instance, to program microglia to prune synapses^59^ or regulate neural stem cell proliferation.^60^ CSF in the dura can also drain through skull channels into the overlying bone marrow, which generates a rich pool of leukocytes that populates the meninges.^61^^–^^66^ Moreover, leukocytes may survey the brain in the CSF following their entry from the blood via the choroid plexus and possibly their movement along the perivascular space.^67^ The architecture of the vasculature, its cellular composition, and its perivascular spaces are diverse across the brain and its bordering tissues, leading to differences in the accessibility to and function of its associated immune cells [for detailed reviews on central nervous system (CNS) tissue immune cell differences see Refs. 68 and 69].

In the past, CSF was assumed to leave the brain either across the cribriform plate along (perineural) olfactory nerve rootlets into lymph vessels or by entering the blood via arachnoid villi and granulations somehow connecting to venous sinuses.^70^^–^^72^ Recent studies, however, suggest instead that CSF is also drained into the dura (possibly via arachnoid granulations) from where CSF enters the meningeal lymphatics, which project to deep cervical lymph nodes^55^^,^^73^^–^^76^ (Fig. 3). However, to date, the mechanism by which CSF crosses the arachnoid barrier into the venous sinus or dural lymphatics remains unknown. In fact, tight junctions between epithelial-like cells comprising the arachnoid should hinder the efflux of CSF and its constituents. Arachnoid granulations have thus been proposed to feature specialized structures for efflux, such as one-way valves or vacuole-forming channels.^77^ Furthermore, the relative contributions of olfactory perineural, venous sinus, and dural lymphatic routes to CSF efflux are unclear and a topic of ongoing discussion.^77^^,^^78^

**Fig. 4:**
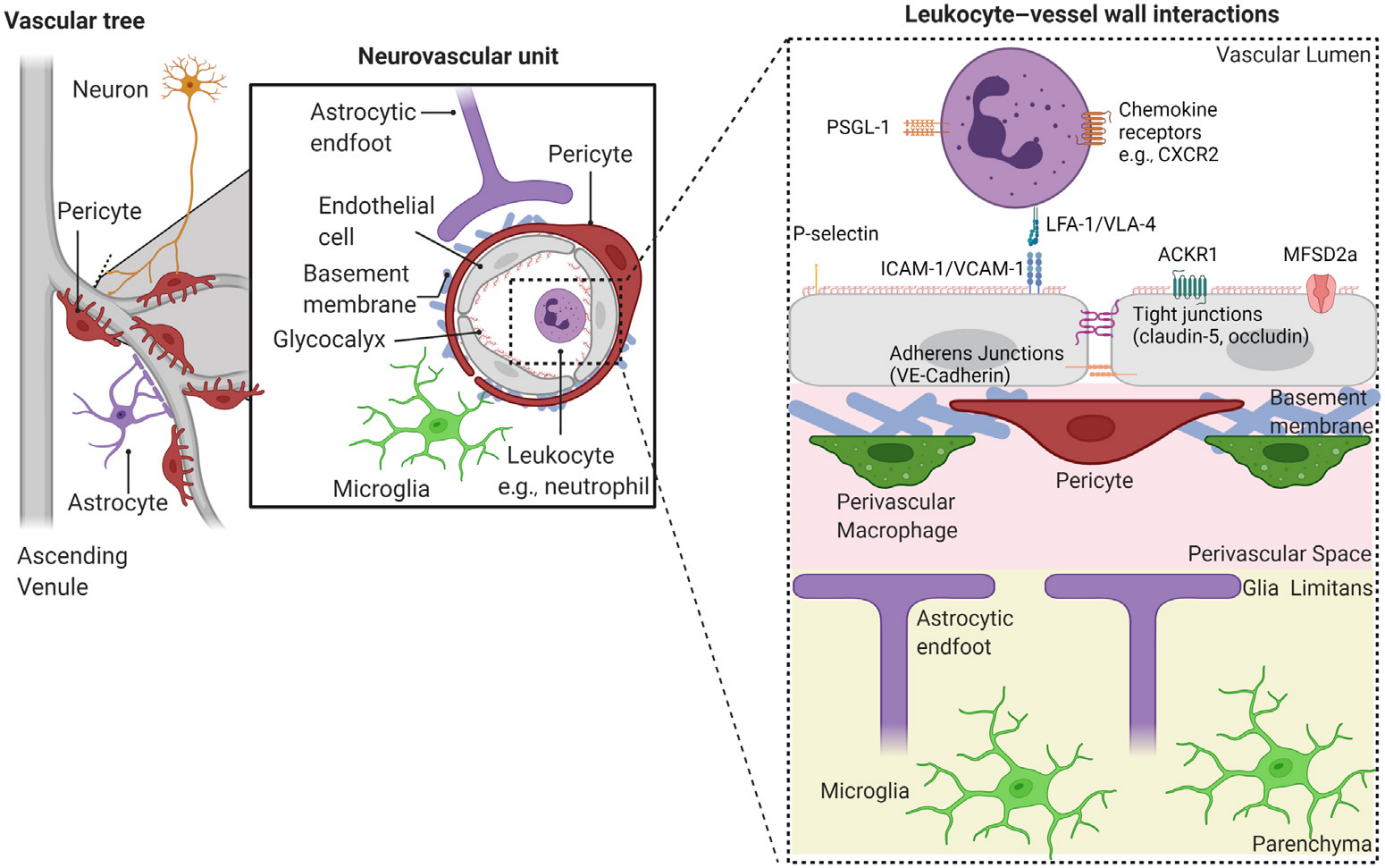
Interaction of leukocytes with, and entry across, the BBB. The neurovascular unit is comprised of a single layer of ECs, contractile pericytes, and smooth muscle cells embedded in the basement membrane, as well as astrocytes and neurons. Neurons and astrocytes send signals to the mural contractile cells to regulate CBF. Perivascular MΦ reside in the perivascular space that is lined with the end-feet of astrocytes forming the glia limitans. Microglia are present in the brain parenchyma but can make direct contact with pericytes via their numerous processes. Infectious agents and tissue damage are detected by these tissue resident immune cells via pattern recognition receptors such as toll-like receptors, which in turn induce transcription and translation of inflammatory cytokines to activate ECs, promoting leukocyte entry and increasing permeability to small molecules. Leukocyte entry occurs across post-capillary vascular segments adjoining ascending venules, where ECs initially upregulate P and E selectin to capture leukocytes in the blood via their respective carbohydrate ligands (e.g., P selectin/PSGL1 interactions). Rolling reduces the speed of leukocytes, allowing prolonged contact time with the endothelium. Subsequent firm adhesive interactions occur via immobilized chemokine ligation to GPCRs on leukocytes, which switches leukocyte integrins into a high affinity state (e.g., ICAM-1/LFA-1 interactions). In the periphery, the majority of leukocyte migration occurs in a paracellular manner, through the junctions of adjacent ECs.^83^ However, in the CNS during exacerbated inflammation T cells can migrate through the body of the EC: a transcellular route^84^ which occurs due to a lack of T cell crawling induced by high ICAM-1 expression.^85^ Additionally, ACKR1, a non-signaling atypical chemokine receptor, mediates the shuttling of chemokines across the BBB, which could also contribute to the mediation of transcellular migration.^86^ It is thought the transcellular route is the preferred mode by which neutrophils move through the brain EC layer.^87^ Approximately 50% of paracellular movement of leukocytes across the vessel wall occurs at tricellular junctions of ECs of the CNS (where three ECs meet).^88^ After crossing the endothelial layer into the perivascular space, leukocytes must then pass the glia limitans layer^89^ to enter the brain parenchyma. Figure created using BioRender.^54^

Peripheral blood leukocytes (Table 1) can also enter the brain across the BBB. Migration occurs across post-capillary vascular segments that connect to AVs. This is a two-step process: passage (1) across the vascular wall into the perivascular space, and subsequently (2) across the glia limitans that comprises the border between the perivascular space and the brain parenchyma.^56^ In the absence of neuroinflammation, leukocyte migration across the BBB is rare, and restricted to activated T cells, which cross the vasculature in an antigen non-specific manner.^79^ T cells need to recognize their cognate antigen from APCs in the perivascular space for them to cross the glia limitans.^80^ Pericytes may also be able to internalize and present antigens to T cells, in addition to stimulating the expansion of and cytokine secretion from T cells already primed by APCs.^81^^,^^82^ During neuroinflammation, however, neutrophils, monocytes, and B cells are also recruited across the BBB into the parenchyma (see Fig. 4). Erythrocytes are also key components of both immunosurveillance and the inflammatory response, by scavenging and sequestering mitochondrial deoxyribonucleic acid (DNA) via toll-like receptor 9 (TLR-9).^90^ During inflammation, the binding of DNA from bacteria, plasmodia, and mitochondria to erythrocyte TLR-9 increases neutrophil infiltration into the spleen, enhances interferon signaling and promotes anaemia by erythrophagocytosis.^91^ Erythrocytes additionally scavenge chemokines, thus regulating chemokine concentrations in the plasma.^92^

### 3.2 Pericyte Control of Leukocyte Trafficking

Recruitment of leukocytes into the brain is a key hallmark of numerous CNS disorders.^93^^,^^94^ For example, neutrophils enter the brain parenchyma early in the progression of AD.^94^^,^^95^ Following stroke, neutrophils may extravasate into the parenchyma^96^^–^^98^ or become trapped and confined within the neurovascular unit.^99^ In addition, the parenchymal infiltration of highly activated T and B cells is a probable driver of early MS pathology.^100^ Upregulation of adhesion molecules by ECs is considered a significant contributing factor to leukocyte influx in CNS disease^95^^,^^101^ and now a role for pericytes in leukocyte recruitment in the CNS is starting to emerge.

Since pericytes embedded in the basement membrane constitute the outermost layer of blood vessels, they are ideally situated to control multiple components of the immune response, as has been highlighted in the periphery. For example, during tumor necrosis factor (TNF) or interleukin (IL)-1β induced inflammation in the murine cremaster muscle, neutrophils are guided via pericyte intercellular adhesion molecule 1 (ICAM-1) to pericyte gaps to breach the vessel wall and enter the interstitium.^102^ In addition, in murine inflamed ear skin, pericytes on capillaries and arterioles aid chemotactic interstitial migration by interacting with neutrophils and monocytes via ICAM-1, and further support neutrophil migratory responses by providing chemokines, including macrophage migration inhibitory factor, C-C Motif Chemokine Ligand 2 (CCL2), and C-X-C Motif Chemokine Ligand 1 (CXCL1).^103^

Intriguingly, while pericytes orchestrate and facilitate leukocyte entry in the periphery, in the CNS it was recently demonstrated that pericytes reduce the permissiveness of the vasculature to leukocyte entry. Mice lacking the PDGFβ retention motif, ${\mathrm{Pdgfb}}^{\mathrm{ret}/\mathrm{ret}}$ (producing a 75% reduction in the number of pericytes in the brain) exhibit an intense influx of leukocytes in health and disease, presumably mediated by loss of pericyte-evoked suppression of ICAM-1 and vascular adhesion molecule 1 (VCAM-1) expression on ECs.^104^^,^^105^ This raises important questions regarding the function of pericytes during leukocyte recruitment in the brain: do brain pericytes prevent leukocyte entry unlike pericytes in other organs such as the cremaster muscle or ear skin, and mechanistically, how is this mediated? Do pericytes physically hinder the recruitment of leukocytes to the interstitium, or do they produce factors involved in maintaining homeostasis to reduce immune cell entry, which may also impact the endothelial cell (EC) phenotype? This has been previously suggested by a study in the mouse retina, whereby depletion of pericytes induced a pro-inflammatory phenotype in ECs, characterized by increased expression of CCL2 or VCAM-1.^106^ In this context, one emerging mediator in pericyte biology is IL-33, which is released via PDGFRβ signaling. IL-33 is considered to be an “alarmin” secreted by a wide range of stromal cells, which polarizes microglia toward an anti-inflammatory phenotype in mouse models of AD.^107^^,^^108^ As such, a lack of pericyte-derived IL-33 could further exacerbate immune cell recruitment and impact EC responses—aspects that remain to be investigated.

Furthermore, pericytes are involved in neuronal control of the CNS immune response, by sensing and relaying signals to neurons early in systemic inflammation. Following systemic lipopolysaccharide (LPS) stimulation in mice, PDGFRβ-expressing pericytes express high levels of the chemokine CCL2 during the initial acute phase of inflammation. Importantly, CCL2 acts on CCR2, the chemokine’s receptor on neurons, and enhances excitatory synaptic transmission.^109^ Given the early release of CCL2 from pericytes in this model, it will be important to define whether this is a transient response and if it is necessary for chronic inflammatory responses. Does pericyte-CCL2 work synergistically with, or independently from, CCL2 produced by microglia? Indeed, CCL2 is also a key factor that recruits monocytes and T cells to sites of inflammation,^110^^,^^111^ suggesting that pericyte-derived CCL2 may also act as a chemotactic molecule in this manner.

Chemokines are presented to leukocytes to facilitate migration into inflamed tissues -a process facilitated in part by the binding of tissue-derived chemokines on the abluminal side of ECs to atypical chemokine receptor 1 (ACKR1), a receptor that facilitates the internalization, transportation, and presentation of chemokines to leukocytes on the luminal aspect of ECs.^92^^,^^112^ For example, in the murine CNS, ACKR1 is upregulated in ECs during inflammation and shuttles chemokines across the BBB to be presented luminally, whereas erythrocyte ACKR1 acts as a chemokine reservoir.^86^ Other members of the ACKR family are involved in chemokine scavenge and degradation.^92^ To the best of our knowledge, no studies have investigated the expression and function of ACKRs on brain pericytes, despite RNAseq data suggesting brain pericytes could express some classes of ACKRs.^14^^,^^16^ Thus, pericytes may play a role in the retention, presentation, or degradation of chemokines via ACKRs, which is yet to be investigated.

In cancer, pericyte deficiency leads to increased leukocyte infiltration in murine experimentally induced tumors, IL-6 upregulation, and hypoxia.^113^ Importantly, the interaction of glioblastoma cells with human pericytes induces expression of anti-inflammatory cytokines IL-10 and TGF-β, which prevent attack of the tumor by host T cell mechanisms, hence promoting tumor survival.^114^^,^^115^ Furthermore, targeting glioma-derived pericytes can increase chemotherapeutic drug effusion into tumors.^116^ Hence, not only do pericytes provide a barrier to leukocyte entry in healthy conditions, but they can propagate tumor growth and malignancy by limiting tumor invasion by the host’s immune system. However, pericytes also play key roles in the vascularization of tumors via angiogenesis,^117^ therefore, suggesting a complicated role for pericytes in cancer.

In summary, it is now clear that pericytes are key mediators in the sensing and propagation of the inflammatory response. However, the role of pericytes seems to be highly organ-specific and dependent on the inflammatory stimulus. Crucially, detailed analysis of pericyte-leukocyte interactions in the CNS is required to understand whether mechanisms are shared between the CNS and the periphery.

## 4 Immune Cell Control of Blood Supply to the CNS

Over 100 years after their discovery, which highlighted their association with the vasculature (see Ref. 118 for a historical overview), microglia are beginning to be recognized as an important modulator of microvascular blood flow in the healthy and diseased adult CNS.^5^^,^^119^^–^^121^ Peripheral immune cells and microglia generate peptides, purines, catecholamines, cytokines, chemokines, and reactive oxygen species (ROS) with established vasoactive properties. These soluble molecules modulate CBF, whether released locally from microglia or invading immune cells, from the perivascular space,^122^ or into the circulation from distal tissues such as the gut.^123^^,^^124^ Immune cells may also alter CBF by scavenging vasoconstrictors such as noradrenaline and endothelin-1 (as occurs in the periphery^125^^,^^126^), degrading matrix proteins regulating vessel stiffness,^127^ contributing to the formation of capillary blocks,^128^^–^^131^ and eliminating capillaries by phagocytosis.^132^^,^^133^ Here, we review some of these emerging roles of immune cells in CBF control.

### 4.1 Microglia form Purinergic Junctions with Capillaries to Control CBF

Microglia can interact with capillaries by sensing purines released from pericytes, ECs or astrocyte endfeet on capillaries and, in pathology, by sensing entry of fibrinogen into the parenchyma from the blood.^134^^–^^136^ The purines, mainly adenosine triphosphate (ATP) and its hydrolysis product adenosine diphosphate (ADP), are sensed by microglial $P2Y_{12}$ receptors ($P2Y_{12}\mathrm{Rs}$).^119^ Microglia are the only cells expressing $P2Y_{12}\mathrm{Rs}$ in the CNS parenchyma,^137^ although in the blood, ADP also activates $P2Y_{12}\mathrm{Rs}$ on platelets, enhancing their aggregation.^138^ Fibrinogen activates microglia by binding to the integrin receptor Mac1 [cluster of differentiation (CD)11b/CD18].^134^ $P2Y_{12}R$ stimulation mediates β1 integrin activation in microglial processes required for chemotaxis toward sites of ATP/ADP released from capillaries, as occurs when brain injury releases ATP from damaged cells and raises ${\left[\mathrm{ATP}\right]}_{o}$.^139^^–^^141^ Interestingly, microglial processes contact 83% of pericytes on capillaries, and cover 15% of the EC surface, predominantly at sites of mitochondria,^120^ which provide ATP released via capillary pannexin 1 (PANX1).^119^ High levels of $P2Y_{12}\mathrm{Rs}$ at the bulbous tips of microglial processes likely facilitate these contacts.^142^ Furthermore, 30% of all microglia somata are closely associated with capillaries.^119^ Microglia also contact capillary segments where astrocyte endfeet are absent, forming an integral part of the glia limitans,^120^^,^^143^^–^^145^ and so are ideally placed to modulate CBF.

Contradictory data exist on the role of microglia in CBF control. In $P2Y_{12}R$ or PANX1 deficient mice, microglia-capillary interactions are reduced, and CBF is increased, suggesting that microglia confer vascular tone by a mechanism dependent on purinergic signaling.^119^ This is consistent with pharmacological microglial depletion increasing capillary diameter and CBF.^119^ Others, however, did not detect changes in CBF upon microglial depletion,^120^ possibly reflecting differences in anaesthetics used or brain regions imaged. Conceivably, CBF changes in $P2Y_{12}R$ or PANX1 deficient mice may also be evoked by reductions in platelet aggregation in these mice.^146^^,^^147^ Furthermore, PANX1 modulates a wide range of physiological functions including inflammasome assembly, dendritic spine development, and sleep-wake cycle patterns,^148^^–^^150^ which may change CBF. Interestingly, global PANX1 KO in mice protects against cerebral infarction in ischaemia,^151^^–^^153^ and this is at least partially mediated by endothelial (but not mural cell) PANX1 KO reducing contractile tone and attenuating leukocyte infiltration.^154^ In contrast, $P2Y_{12}R$ blockade increases infarct size and microglial elimination reduces CBF after experimental stroke in murine models.^39^^,^^120^ Microglial depletion, $P2Y_{12}R$ knock-out, or pharmacological $P2Y_{12}R$ blockade in mice also reduce neuronally evoked increases in CBF by ∼16%,^120^ suggesting that microglia may contribute to neurovascular coupling (NVC). Importantly, $P2Y_{12}R$ expression is reduced in various neurological diseases as shown in humans in AD and MS and in murine models of ischemic stroke,^155^^–^^157^ which may therefore alter microglia-capillary interactions^132^^,^^135^^,^^158^ and impair NVC^159^^–^^161^ in these diseases.

In summary, these studies suggest a role for microglia-mediated CBF control beyond that of vessel growth during development^162^^–^^164^ (Fig. 1) and warrant further research into the mechanisms by which PANX1 and $P2Y_{12}\mathrm{Rs}$ control CBF.

### 4.2 Immune Cells Signal to Mural Cells Directly via the Blood or CSF and Block Capillaries in Disease

Microglia and cerebral perivascular macrophages (PVMs) signal directly to mural cells to modulate CBF. This is facilitated by their close association with mural cells. Unlike microglia, however, PVMs lack ramified processes and $P2Y_{12}\mathrm{Rs}$, and are located outside the glia limitans and within the perivascular space, closely juxtaposed to PAs and capillaries of at least the first to third branching order^122^^,^^165^ (Fig. 1). Peripheral immune cells also regulate mural cell contractile tone, although probably on a slower timescale, by releasing signaling molecules into the blood or CSF.^123^^,^^166^^–^^168^ Molecules released into the blood may act on ECs at the luminal side of the BBB, whereas those in the CSF may modulate vascular tone by entering the brain through the glia limitans at the pial surface or by being transported along perivascular and glymphatic routes (Figs. 1 and 3). Importantly, the glia limitans, arachnoid mater, and dural vasculature allow the passage of molecules up to ∼40  kDa in size (e.g., 40-kDa dextran or HRP),^25^^,^^169^^–^^171^ which is larger than the size of most cytokines (∼5 to 25 kDa). Leukocytes in the meninges may therefore release molecules into the CSF to modulate CBF.

In the healthy murine brain, circulating leukocytes stall transiently in 0.4% of cerebral capillaries, possibly by interacting with the glycocalyx or selectins on ECs^128^^,^^172^^–^^174^ (Fig. 4). However in disease, disruption of the glycocalyx, upregulation of selectins and adhesion molecules or any narrowing of the capillary lumen evoked by pericyte contraction or oedema may cause leukocytes, which are less distensible and larger than erythrocytes, to become trapped in CNS capillaries. Neutrophil blocks were shown to occur in rodent models of stroke, diabetic retinopathy, sepsis, cerebral malaria, AD, and subcortical vascular dementia.^128^^–^^131^^,^^175^^–^^180^

**Fig. 5:**
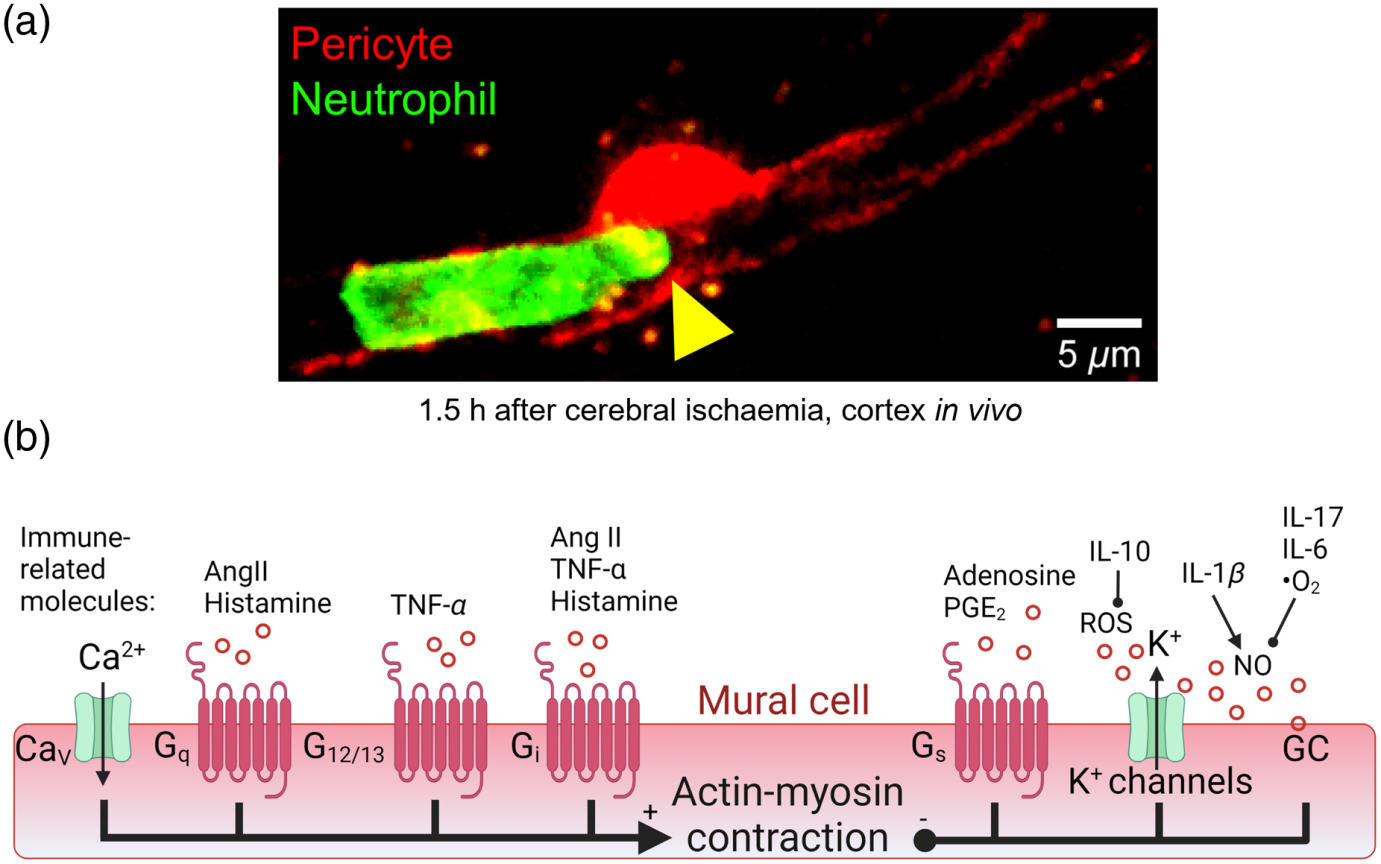
Mural cell leukocyte interactions modulating blood flow. (a) *In vivo* two-photon imaging of a neutrophil stalled near a pericyte soma in a cerebral capillary 1.5 h after bilateral common carotid artery occlusion in an adult mouse expressing dsRed under the pericyte NG2 promoter. Blood neutrophils are labeled using an antibody conjugated to Ly6G (0.1  mg/kg i.v.). Arrow indicates the narrowed lumen diameter at the pericyte soma. (b) Major ion channels and GPCRs modulating mural cell contractile tone in response to immune molecules. Stimulation of GPCRs coupled to $G_{q}$, $G_{12/13}$, and $G_{i}$ and ${\mathrm{Ca}}^{2+}$ influx through ${\mathrm{Ca}}_{V}s$ promotes contraction, whereas GPCRs coupled to $G_{s}$, $K^{+}$ efflux and GC activity reduce contraction. Immune cells may modulate contractility by releasing angiotensin II (Ang II), TNF-α, adenosine, $\text{P}{\mathrm{GE}}_{2}$, and NO. NO generated by nitric oxide synthase (NOS) activates mural cell GC, which promotes vasodilation by enhancing the activity of MLCP. NO levels are increased by IL-1β and reduced by IL-17 and IL-6 negatively regulating eNOS and by ${•O}_{2}$ reacting with NO. Figure created in part using BioRender.^54^

Similar vascular blocks by leukocytes can be observed in patients with AD^95^ and stroke,^99^ although the human leukocyte blood composition is remarkably different from that of mice (Table 1), in that there are more neutrophils and less lymphocytes (Table 1). In stroke patients, live computed tomography imaging of granulocytes labeled with a radioactive tracer revealed that granulocyte accumulation in regions of cerebral infarction correlates with worse neurological outcome and infarct volume.^181^^,^^182^ Since ischaemic stroke or the toxic build-up of Aβ oligomers in AD leads to pericytes constricting capillaries near their somata, where most circumferential processes are located,^5^^,^^8^ leukocytes may become trapped near pericyte somata as suggested by our data in a rodent model of stroke [Fig. 5(a)].^183^ Formation of capillary blocks may also be enhanced by neutrophils aggregating with platelets, possibly by generating extracellular DNA traps as occurs in arterial clots of ischemic stroke patients^184^^,^^185^ or by the release of inflammatory molecules as described below.

### 4.3 Modes of Mural Cell Contraction by Immune Molecules

There are numerous immune-related molecules with known effects on blood flow. They include adenosine, angiotensin II (Ang II), prostaglandin $E_{2}$ (${\mathrm{PGE}}_{2}$), IL-1α, and nitric oxide (NO) generated by microglia,^120^^,^^121^^,^^186^^–^^188^ histamine produced by mast cells,^189^^–^^194^ fractalkine (FKN) released by neurons,^121^ IL-17 generated by T helper cells,^123^ TGF-β released from astrocytes,^195^^,^^196^ IL-10 released from T regulatory cells^197^ and ROS, IL-1α, IL-1β, IL-6, and TNFα generated by various immune cells (Table 2).

**Table 2:** Possible pathways mediating immune cell regulation of vascular tone in health and disease.

Expected vasomotor effect	Generating cell type	Agonist	Mechanism	References
Vasodilation or increased blood flow	Microglia	NO	Stimulates mural cell GC	188,198
		adenosine	$G_{s}\mathrm{PCR}$-coupled adenosine receptors on mural cells	40,120,199
		${\mathrm{PGE}}_{2}$	$G_{s}\mathrm{PCR}$-coupled EP4 receptors on pericytes and $G_{q}$-coupled endothelial EP1 receptors	11,14,186,200
		IL-1β	IL-1 receptor mediated release of NO and ${\mathrm{PGE}}_{2}$	141,199,201–205
		IL-1α	Pial artery dilation	187
	Microglia, perivascular MΦ, blood and meningeal leukocytes	ROS	ATP-gated and ${\mathrm{Ca}}^{2+}$-activated $K^{+}$ channels in SMCs	165,206–209
	T regulatory cells	IL-10	Reduces Ang II evoked release of $•O_{2}$–	197
	Mast cells	histamine	Capillary dilation	191
	Various leukocytes	TNFα	Increases blood flow by stimulating NO release	210
Vasoconstriction or decreased blood flow	Microglia	Thromboxane-A2	$G_{q}\mathrm{PCR}$-coupled thromboxane A2 receptors on mural cells	7,14
		IL-1β	Inflammation and leukocyte infiltration	203,211
		Angiotensin II	$G_{i}$ and $G_{q}$-coupled Ang II type 1 receptors on mural cells	121
	Microglia, PVM, blood and meningeal leukocytes, pericytes	ROS	${\mathrm{Ca}}^{2+}$ release from stores in pericytes; reduces NO bioavailability; stimulates release of endothelin-1 activating Gq-coupled ${\mathrm{ET}}_{A}$ receptors on pericytes	165,206–208,212–214
	Neurons	FKN	Stimulates microglia to release Ang II acting on Ang II type 1 receptors on mural cells	121
	Gut T helper cells	IL-17	Negatively regulates eNOS by enhancing its Thr495 phosphorylation	123,124
	Various leukocytes	IL-6	Negatively regulates eNOS by inhibiting its phosphorylation at Ser1177	215,216
	Mast cells	histamine	Depolarizes and contracts pericytes via histamine ($H_{1}$) receptors	189,192–194
	Astrocytes	TGF-β	Excessive production reduces CBF possibly by inducing mural cell loss	195,196

There are established mechanisms by which these immune molecules may modulate the contractile tone of pericytes and SMCs [Fig. 5(b)]. Molecules may directly act on mural cells or signal through neighboring cells such as ECs to modulate the activity of mural cell ion channels, guanylyl cyclase (GC), or G-protein coupled receptors (GPCRs). GPCRs coupled to $G_{q/11}$, $G_{s}$, $G_{i/o}$, or $G_{12/13}$ α subunits can influence a wide range of ${\mathrm{Ca}}^{2+}$-, ${\mathrm{Cl}}^{-}$-, ${\mathrm{Na}}^{+}$-, or $K^{+}$-permeable surface membrane ion channels to regulate mural cell contractile tone. The surface membrane ion channels involved can be broadly divided into those having a hyperpolarizing or depolarizing influence on surface membrane potential ($V_{m}$). In addition there are ${\mathrm{Ca}}^{2+}$-permeable channels in internal stores that are gated by inositol trisphosphate (${\mathrm{IP}}_{3}$) generated by $G_{q/11}$ activating phospholipase C.

Depolarization evokes ${\mathrm{Ca}}^{2+}$ influx from the extracellular milieu via voltage-gated ${\mathrm{Ca}}^{2+}$ channels (${\mathrm{Ca}}_{V}s$). This ${\left[{\mathrm{Ca}}^{2+}\right]}_{i}$ rise causes ${\mathrm{Ca}}^{2+}$ to bind to calmodulin (CaM). The ${\mathrm{Ca}}^{2+}$-CaM complex then activates myosin light chain kinase (MLCK), which in turn phosphorylates the myosin light chain (MLC) of myosin II causing actin-myosin crossbridge cycling and contraction [Fig. 5(b)]. In mural cells, $G_{q}\mathrm{PCR}$ agonists activate MLCK by raising ${\left[{\mathrm{Ca}}^{2+}\right]}_{i}$. Hyperpolarization, on the other hand, is primarily evoked by $K^{+}$ efflux, for instance, via inward rectifier $K^{+}$ (Kir2.1 and Kir2.2) or ATP-sensitive $K^{+}$ ($K_{\mathrm{ATP}}$; Kir6.1) channels,^14^^,^^217^^–^^219^ causing voltage-gated calcium channels to close and vessels to dilate.

Signaling via GC or GPCRs coupled to $G_{s}$, $G_{i/o}$, or $G_{12/13}$ modulates the activity of myosin light chain phosphatase (MLCP), which dephosphorylates MLC to reduce contraction. While GC and $G_{s}\mathrm{PCRs}$ enhance MLCP activity by facilitating cAMP production via adenylate cyclase (AC), $G_{i}\mathrm{PCRs}$ and $G_{12/13}\mathrm{PCRs}$ have an opposing effect; they negatively regulate MLCP by inhibiting AC or stimulating rho-associated protein kinase, respectively, thus evoking contraction.^220^ $G_{s}\mathrm{PCR}$ agonists such as adenosine can also hyperpolarize mural cells by evoking $K^{+}$ efflux via $K_{\mathrm{ATP}}$ channels.^221^

### 4.4 Microglia and Peripheral Immune Cells Release Vasoactive Molecules to Control Blood Flow

Microglia generate vasodilating adenosine by hydrolyzing ATP using the membrane-bound ectoenzymes CD39 and CD73.^39^^,^^40^ This may facilitate capillary dilation, at least in conditions of hypercapnic ${\mathrm{CO}}_{2}$ challenge in mice,^119^^,^^120^ and it may also contribute to adenosine-evoked increases in CBF during NVC.^222^ Adenosine relaxes pericytes via $G_{s}$-coupled adenosine receptors [Fig. 5(b)]. In contrast, ATP and ADP contract pericytes by stimulating P2X receptors (ATP-gated channels mediating cation influx) and $G_{q}$-coupled ${P2Y}_{1}$ and ${P2Y}_{2}$ receptors (promoting ${\mathrm{Ca}}^{2+}$ release from stores), respectively.^223^^–^^227^ ATP hydrolysis by microglia may thus attenuate contractile tone.

Microglia can also produce the prostanoid ${\mathrm{PGE}}_{2}$ via cyclooxygenase (COX)^14^^,^^186^ and IL-1β via the NLRP3 inflammasome.^141^^,^^201^ ${\mathrm{PGE}}_{2}$ evokes capillary dilation by stimulating $G_{s}$-coupled ${\mathrm{EP}}_{4}$ receptors on pericytes and $G_{q}$-coupled endothelial EP1 receptors,^11^^,^^200^ whereas IL-1β activates its receptors predominantly on ECs, which increases CBF by promoting the release of NO.^202^^–^^205^ However, prolonged IL-1β application was found to enhance hypoperfusion,^211^ likely by promoting inflammation and leukocyte infiltration.^203^

Since microglial ${\left[{\mathrm{Ca}}^{2+}\right]}_{i}$ transients are partly driven by the activity of neurons^228^ and regulate COX, and NLRP3 and matrix metalloproteinase (MMP)-mediated cleavage of membrane-bound cytokines into their soluble forms,^229^^–^^233^ it is conceivable that neurotransmitters modulate microglial-evoked changes in CBF. Indeed, the factors controlling the release of various vasoactive prostanoids (via COX) and cytokines (via NLRP3 or MMPs) are largely unexplored in immune cells. Transcriptome studies, for instance, suggest that microglia are uniquely endowed^14^ with the enzyme thromboxane $A_{2}$ synthase (downstream of COX) that generates the prostanoid thromboxane $A_{2}$, which contracts pericytes, whereas the cytokine IL-1α is generated in microglia and evokes dilation of pial arteries.^7^^,^^14^^,^^187^

Neurons also signal to microglia to alter blood flow via the chemokine fractalkine (FKN/CX3CL1). FKN is predominantly present in neurons (membrane-bound or released in a soluble form) and binds to CX3CR1 selectively expressed in microglia or MΦ.^234^ In the healthy brain, FKN promotes synaptic strength, neurogenesis, and memory formation and may act as a “find me” signal for microglia to clear neuronal debris,^201^^,^^235^^,^^236^ but, after stroke, signaling via FKN reduces CBF, enhances neuronal apoptosis and worsens neurological outcome in mice.^120^^,^^237^^,^^238^ In the mouse retina, application of soluble FKN (mimicking its release from damaged neurons^239^) evokes rapid capillary constriction, possibly by a mechanism involving microglial release of Ang II acting on $G_{i}$ and $G_{q}$-coupled Ang II type 1 receptors.^121^ Furthermore, our unpublished data show that FKN contracts pericytes and decreases blood flow in the murine cerebral cortex. Blocking CX3CR1 may thus provide a therapeutic approach to reduce the contraction of pericytes that contributes to the no-reflow of blood in capillaries after stroke.^8^^,^^206^^,^^240^

ROS generated by NADPH oxidase (NOX2) in immune cells can be released in the brain by microglia, PVMs or infiltrating leukocytes,^165^^,^^207^^,^^208^ in the blood by circulating leukocytes such as neutrophils (e.g., after stroke in humans^241^) or from the meninges into the brain (e.g., after traumatic brain injury^170^). ROS directly modulate the contractile tone of pericytes and SMCs; in pericytes by stimulating ${\mathrm{Ca}}^{2+}$ release from stores (possibly via endothelin-1 release^5^), which raises ${\left[{\mathrm{Ca}}^{2+}\right]}_{i}$ and enhances vasoconstriction^206^^,^^212^ and in SMCs by activating ATP-gated and ${\mathrm{Ca}}^{2+}$-activated $K^{+}$ channels, which hyperpolarize $V_{m}$ and evoke vasodilation.^206^^,^^209^ ROS release following ischemic stroke induces pericyte contraction and contributes to capillary no-reflow, which can be partially reversed with a NOX2 blocker.^206^

Superoxide ($•O_{2}$) generated by NOX2 can also react with endothelial-derived NO to form peroxynitrite, thus reducing NO bioavailability.^213^ Indeed, NO-mediated increases in CBF evoked by neuronal activity are impaired by PVMs producing $•O_{2}$, for instance, in response to Ang II binding to AT1 receptors^214^ or Aβ activating the innate immunity receptor CD36.^122^ Aβ also evokes release of ROS from pericytes (via NOX4) and from microglia (via NOX2), causing pericyte contraction by a mechanism dependent on downstream release of endothelin-1 activating $G_{q}$-coupled ${\mathrm{ET}}_{A}$ receptors.^5^ ROS and endothelin-1 are also thought to contribute to cerebral hypoperfusion in patients with MS.^242^^,^^243^ The anti-inflammatory cytokine IL-10 released by T regulatory cells largely prevents the Ang II evoked release of $•O_{2}$ and restores NVC,^197^ which is consistent with the cerebroprotective function of IL-10 after stroke in mice.^244^

In mice on a high salt diet, gut T helper (TH17) cell release of IL-17 into the circulation reduces the activity of eNOS, which reduces CBF by 25%.^123^^,^^124^ Elevated levels of IL-17 found in the blood of patients with AD, MS and stroke^245^ and infiltrating neutrophils releasing IL-17 in the cortex of AD mice^95^ may reduce endothelial NOS (eNOS) activity in a similar manner. Furthermore, elevated plasma levels of IL-6, which promotes coagulation and negatively regulates eNOS (by inhibiting its phosphorylation at Ser1177),^215^^,^^216^ were associated with CBF decreases in aging individuals, stroke patients, and in patients recovered from COVID-19.^246^^–^^248^

Importantly, systemic infection evoked decreases in CBF may enhance cognitive decline in patients. For instance, 62% of hospitalized COVID-19 patients present with a reduction in brain energy supply, most commonly, in the form of an ischemic stroke^249^ and 34% show neurological and psychiatric deficits in the six months following COVID-19 infection.^250^ Cerebral ischemia evoked by capillary constriction is thought to be a major cause of brain injury in patients with sepsis,^251^^–^^254^ which occurs when the immune system excessively generates cytokines in response to infection. A similar “cytokine storm” occurs in severe COVID-19 patients^255^ although, similar to human immunodeficiency virus (HIV), SARS-CoV-2 induces profound lymphopenia, in particular a reduction in CD4+ and CD8+ T cells.^256^^,^^257^ Notably, a decline in CD4+ T cells in HIV patients or in a macaque model of AIDS correlates with a decrease in CBF.^258^^,^^259^ Although respiratory failure is a major risk factor for cerebral ischemia in COVID-19 patients, SARS-CoV-2 also impairs the cerebral vasculature directly by inducing pericyte contraction,^260^ EC death,^261^ and microthrombi formation.^262^ Systemic infection also exacerbates cerebral hypoperfusion in AD patients, presumably by inducing the release of cytokines,^263^ which is expected to accelerate cognitive decline in these patients.^264^ Interventions aimed at restoring CBF following severe infection may thus help to improve cognition in these patients.

## 5 Do Immune Cells Contribute Significantly to the Maintenance and Disruption of the Blood–Brain Barrier?

The BBB is a highly specialized vascular barrier that limits the influx of serum proteins, leukocytes, and toxic substances such as glutamate and ATP into the brain parenchyma and pumps out waste—functions that are primarily mediated by ECs, pericytes, and astrocytes (Fig. 4). The BBB results from: (1) transendothelial tight junctions between ECs that confer a high resistance to paracellular diffusion of solutes, (2) suppression of endocytic vesicle-mediated transcytosis of macromolecules such as proteins and peptides, and (3) solute carriers that transport carbohydrates, vitamins, amino acids, hormones, monocarboxylic acids, and nucleotides.^2^^,^^265^ CNS ECs exhibit a BBB-specific gene expression profile, with similar core changes in gene expression being observed across different CNS disorders.^266^ This specialized layer of ECs is held together by tight junctions (e.g., occludin and claudin, which link the cytoskeleton through scaffolding proteins such as zonula occludens-1) and adherens junctions (cadherins, which connect intracellular actin filaments via α, β, and γ catenins).^267^ The BBB maintains low levels of permeability-enhancing proteins on ECs including the Tie2 ligand angiopoietin-2 and the plasmalemma vesicle-associated protein, which is required for endothelial vesicle trafficking. Expression of various leukocyte adhesion molecules (ICAM1, VCAM1, activated leukocyte cell adhesion molecule (ALCAM), and galectin-3) are also suppressed. This is to limit leukocyte influx, which can contribute to BBB leakiness caused by disruption of EC junctional molecules and the extracellular matrix via leukocyte release of (1) pro-inflammatory cytokines, such as TNF,^268^ (2) ROS,^269^^,^^270^ or (3) MMPs.^271^^–^^275^ These leukocyte-derived molecules are also able to induce neurotoxicity.^276^

In the brain, ECs show a lower rate of transcytosis than ECs of any other organ.^277^ This is in part because the major facilitator superfamily domain—containing 2a (Mfsd2a) protein, a sodium-dependent lysophosphatidylcholine transporter—supresses transcytosis in capillaries (but notably not in arterioles^278^) because lipids transported by MFSD2a establish a unique lipid composition of CNS EC plasma membranes that inhibits endothelial caveolae vesicle formation.^279^^,^^280^ With age, MFSD2a becomes downregulated, whereas caveolin vesicle density is increased, in association with reduced pericyte coverage and increased BBB permeability.^15^ In addition, the level of MFSD2a is reduced upon LPS exposure, suggesting its expression can be modulated by inflammation.^281^ Interestingly, lymphocyte transcellular migration (migration through the EC body) relies on the translocation of ICAM-1 to caveolae-rich domains of ECs, to create a path through which the lymphocyte can migrate,^282^ and ECs, which exhibit low MFSD2a levels have high ICAM-1 expression.^283^ Thus, the same factors regulating transcytosis of molecules may also influence transcellular migration of lymphocytes.

Key to the development and maintenance of the BBB are pericytes, which are essential for the development of tight junctions between ECs,^105^^,^^284^^,^^285^ the expression of MFSD2a in ECs^280^ thus suppressing permeability-enhancing proteins,^104^^,^^105^ the stabilization of tight junctions by secretion of angiopoietin-1,^286^ and inducing growth of the endothelial tube, which is suggested to widen capillary diameters near pericyte somata.^5^^,^^8^ Pericyte deficiency in mutant mice (with low levels of PDGFRβ or lacking the PDGF-BB retention motif) severely impairs the BBB. This results in an influx of toxic blood-borne solutes (e.g., cytokines and serum proteins), impaired NVC,^9^^,^^284^ disrupted expression of leukocyte adhesion molecules,^105^ activation of microglia, and damage to neurons.^285^ Accordingly, in AD patients’ brains, pericyte loss correlates with enhanced BBB leakage.^287^ Additionally, increased soluble PDGFRβ in the CSF correlates with increased measures of BBB dysfunction and cognitive impairment,^288^^,^^289^ highlighting the key importance of this cell type for maintaining a healthy BBB and avoiding pathology. Activation of the CypA-MMP9 pathway in pericytes (e.g., in ApoE4 carriers) can contribute to BBB breakdown, highlighting how crucial this cell type is for barrier function.^290^^,^^291^

Ablation of microglia in the healthy murine CNS does not compromise BBB integrity.^103^^,^^292^^–^^294^ However, microglia do play a key role in the rapid repair of the vasculature upon BBB impairment. Specifically, in a manner dependent on ADP-sensing $P2Y_{12}$ receptors, microglia extend processes toward, and aggregate at, sites of vascular damage (as induced by laser injury) and seal the broken vessel using a mechanism dependent on E-cadherin.^295^ In addition, microglia may also secrete trophic factors to encourage endothelial growth.^295^ This phenomenon has been further explored in zebrafish with respect to MΦ, where these cells physically repair vessels by pulling two endothelial ends together in a microfilament-dependent manner.^296^ Thus, immune cells can repair damage by providing adhesive molecules and by physical traction.

Recent data also suggest that microglia play dual roles in the regulation of the BBB, dependent on the timing of the inflammatory response. Initially, following LPS challenge in mice, microglial contact with cerebral blood vessels protects BBB integrity, by increasing expression of the tight junctional protein claudin-5,^297^ a phenomenon that has also been shown to be mediated by astrocytes.^298^ However, prolonged inflammation results in a more activated microglial phenotype, resulting in phagocytosis of astrocytic end-feet and BBB integrity loss.^297^ In mild hypoxia in the spinal cord, microglial depletion enhances tight junction loss and BBB permeability.^293^ In the brain following stroke in mice, microglia migrate toward the hypoxic vasculature and, in principle, aid repair by phagocytosing and clearing damaged sites. However, this leads to a positive feedback loop, where the vasculature becomes leaky to the blood serum components albumin and fibrinogen, which upregulates expression of inflammatory cytokines, and promotes further recruitment of microglia.^132^ Indeed, deposition of fibrinogen in the parenchyma as a consequence of BBB dysregulation in mouse models of MS induces vascular microglia clustering, contributing to neuronal damage.^135^ Therefore, while the primary role of microglia is protective, the consequences of their actions can be damaging to the parenchyma, as occurs following ischaemia and in AD patient brains.^299^ These studies highlight that the role microglia play, whether protective or destructive, is very much dependent on the type of injury, and the time frame.

While microglia play key roles in the regulation of the BBB, peripheral leukocytes predominantly contribute to BBB breakdown during pathology. These actions can be mediated by ROS and MMP release, which damage EC junctions, for example by interfering with the β-catenin complex.^300^ Neutrophils are rich sources of these mediators, and contribute to the breakdown of the BBB in various neurological pathologies,^95^^,^^301^^,^^302^ and T cells have also been shown to mediate damage in this manner.^303^ Infiltrating T cells can perturb the BBB via the action of cytokines IL-17 and IL-22,^304^ which release ROS from ECs, disrupting tight junctions.^305^

In addition, prolonged stalling of neutrophils in vessels may impair the integrity of the BBB by enhancing endothelial actin depolymerization and the breakdown of adherens junctions between ECs, as occurs in the periphery.^306^ More recently, neutrophil extracellular traps (NETs) have been observed to form both intravascularly and extravascularly during neurological disease, which may contribute to BBB permeability increases.^95^^,^^302^ NETs are structures formed by highly activated neutrophils that extrude DNA and intracellular contents to capture pathogens, degrade bacterial toxic factors, and kill bacteria.^307^ Neutrophils from mice subjected to stroke are more likely to form NETs, and removal of NETs reduces BBB leakage and improves pericyte coverage.^302^ The mechanism by which NETs induce BBB breakdown is not yet clear, but since NETs expose and spill intracellular content (histones, and granule content including proteases), it is likely that proteases and ROS contribute.

In summary, microglia can play protective and detrimental roles in the regulation of the BBB, but peripheral blood leukocytes are predominantly destructive in pathology.

## 6 Summary and Possible Therapeutic Avenues

Immune–vascular interactions are a therapeutic target for various neurological diseases. CBF decreases occurring after stroke or early in the progression of AD could, for instance, be therapeutically targeted by inhibiting FKN receptors or blocking ROS production, respectively.^5^^,^^8^^,^^120^^,^^308^^–^^310^ This may reduce the plugging of capillaries by blood leukocytes, improve energy supply, and reduce capillary pericyte and neuronal loss in later stages of disease.^288^^,^^311^ Pericyte degeneration also occurs early in human MS, and pericyte-deficiency in an MS mouse model is lethal due to excessive immune cell influx.^104^^,^^312^ A possible therapeutic strategy may thus aim to restore pericyte coverage to improve barrier function in these diseases.

Another clinical approach could involve recruiting immune cell subsets with protective properties to the diseased brain. Promising murine studies highlight that recruitment of regulatory T cells improves cognitive function in AD,^313^^,^^314^ promotes microglia-mediated oligodendrogenesis following stroke,^315^ prevents Ang II evoked disruption of NVC^197^ and ameliorates neutrophil MMP-9 mediated BBB breakdown.^316^ Furthermore, augmenting immunosurveillance of the brain by enhancing lymphatic drainage (promoting antigen exposure to immune cells) facilitates the CD8+ T cell mediated clearance of brain tumors.^317^ A similar approach could be adopted to manipulate pericytes to allow drug and immune cell access specifically to tumors.

In conclusion, immune–vascular interactions play both homeostatic and pathogenic roles in the CNS depending on the context and injury. This field is currently at an exciting stage, where future work will identify new therapeutic avenues to help combat CNS disease.
